# Physical exercise can enhance meaning in life of college students: the chain mediating role of self-efficacy and life satisfaction

**DOI:** 10.3389/fpsyg.2023.1306257

**Published:** 2024-01-11

**Authors:** Yuncheng Guo, Keyi Qin, Yida Yu, Lixia Wang, Fanglei Xu, Qishan Zheng, Xinyu Hou, Yan Zhang, Biying Hu, Qingping Hu, Chuanhua Gu, Jianhong Zheng

**Affiliations:** ^1^School of Physical Education and Sports, Central China Normal University, Wuhan, China; ^2^School of Psychology, Central China Normal University, Wuhan, China; ^3^Faculty of Education, University of Macau, Macau, China; ^4^Campus Hospital, Central China Normal University, Wuhan, China; ^5^Department of Psychology and Key Laboratory of Psychological Assessment and Rehabilitation for Exceptional Children / Guangdong Provincial Key Laboratory of Development and Education for Special Needs Children, Lingnan Normal University, Guangdong, China

**Keywords:** physical exercise, life satisfaction, self-efficacy, meaning in life, chain mediating role, college students

## Abstract

Meaning in life refers to an individual’s capacity to understand and grasp the meaning of their own existence, as well as being aware of the goals, tasks, or missions in their personal life. Previous studies have found that college students lack meaning in life, but physical exercise can enhance it. In this study, 3,196 college students completed self-report questionnaires to assess self-efficacy, life satisfaction, physical exercise, and meaning in life. The results revealed that the physical exercise not only influenced an individual’s perceived meaning in life directly, but also influenced it through self-efficacy. Furthermore, it confirmed the chain mediating role of self-efficacy and life satisfaction, whereby engaging in physical exercise can ultimately impact meaning in life through self-efficacy and life satisfaction. This discovery can help educators create interventions to improve college students’ physical exercise engagement and overall life satisfaction.

## 1 Introduction

It is important to be educated about life. Not understanding life can have serious consequences, including the extinction of life in various forms. According to the World Health Organization’s survey in 2019, suicide is the fourth leading cause of death in the 15–29 age group worldwide (World Health Organization, 2021). Due to the significance of mental health in recent years, suicide among college students has become a research hotspot. Psychologists have warned about the negative effects of lacking meaning in life, which can provide individuals with a sense of personal importance. Irvin Yalom, author of Existential Psychotherapy, states that almost all of his patients believe there is “no meaning in life” (Glaw et al., 2017). According to Carl Jung, one-third of the psychopaths he encountered did not have neurosis but were diagnosed with mental disorders because they had a sense of life’s futility (Glaw et al., 2017). Previous studies have shown that a lower meaning in life is linked to both psychological distress and physical illness, leading to a decline in normal physical and mental functions in individuals (Dunn and O’Brien, 2009; Steger et al., 2009a,b). Believing in the purpose and meaning of life is an important aspect of mental health, according to Galek et al. (2015). This suggests that perceiving meaning in life can positively impact an individual’s mental well-being and help them appreciate the value of life. According to a survey conducted by Li and Lu (2010), more than 50% of Chinese college students reported feeling a lack of purpose in life. Studies claimed that when college students feel that their lives lack meaning, they may experience a concerning condition known as “noogenic neurosis” (Frankl, 1985). This can lead to a decreased zest for life, a lack of motivation to plan for the future, and may even result in negative emotions like anxiety, depression, fear, and feelings of inadequacy. In severe cases, it can lead to psychological problems, and even raise behavioral tendencies such as suicide, violence and abuse (Zika and Chamberlain, 1992).

Instead, college students who have a strong meaning in life tend to have a positive and optimistic outlook towards their work and goals. This mindset not only helps them adjust to college life more easily but also shields them from physical and mental health issues and lowers the likelihood of self-harm and suicide (Makola, 2014). The concept of finding meaning in life stresses the significance of having goals and centers on how each person creates, understands, and goes after their own purposes (Steger et al., 2006), this aligns with the benefits of engaging in physical exercise, which can motivate individuals to pursue their goals and give them a sense of personal mission in life. Moreover, several studies have confirmed that physical exercise positively impacts psychological and social adaptation (Hooker and Masters, 2018; Schuch et al., 2018; Yemiscigil and Vlaev, 2021). However, few studies have explored how physical exercise relates to meaning in life of college students (Hooker et al., 2018). Therefore, it is currently unclear how physical exercise may affect one’s meaning in life.

To sum up, improving the meaning in life among college students has significant benefits for their psychological well-being and social adjustment. It is essential to explore potential mechanisms for both theoretical and practical purposes. This study aims to explore the mediating mechanisms underlying the relationship between physical exercise and meaning in life.

### 1.1 Physical exercise and meaning in life

The concept of meaning in life pertains to how one perceives and comprehends the purpose of their existence, as well as their awareness of the goals, tasks, or missions they wish to fulfill (Steger et al., 2006), including having meaning (referring to the degree to which an individual feels whether his life is meaningful) and seeking meaning (referring to the degree to which an individual actively seeks the meaning of life). However, college students’ common occurrence of suicide reveals their insufficient perception of life’s meaning (Li and Lu, 2010).

According to American College of Sports Medicine (1991), physical exercise involves planned, organized, and repetitive activities that boost cardiorespiratory function. It is a crucial factor in maintaining good mental and physical health. One potential way to enhance an individual’s meaning in life is by increasing physical exercise. On the one hand, physical exercise has theoretical support for improving emotional adaptation. The theory of mood-enhancing benefits of exercise proposes that physical exercise can improve mood by reducing negative emotions such as anxiety, depression, and pressure, as well as their corresponding behavioral effects (Anderson and Brice, 2011; Ruby et al., 2011). Additionally, it is believed to help prevent the development of emotional disorders, such as anxiety and depression (Rebar et al., 2015). Exercise psychology also suggests that physical exercise enhances positive emotions and vitality, leading to improved mental health (Rebar et al., 2015). On the other hand, relevant empirical studies further support the above views. A study showed that physical exercise improves meaning and quality of life for older adults (Takkinen et al., 2001). Furthermore, a study conducted on college students showed that regular physical exercise can positively predict their meaning in life (Liu, 2012; Xia et al., 2023). Based on the above analysis, this study put forward hypothesis 1: Physical exercise is positively associated with meaning in life of college students.

### 1.2 Relationship between self-efficacy, life satisfaction, and meaning in life

Self-efficacy refers to an individual’s personal evaluation of their ability to perform a specific behavior or master a particular capacity in a given situation. It is commonly considered as a predictor of motivation and can explain corresponding behaviors. Self-efficacy also serves as a psychological motivation for individuals to continuously regulate themselves [as stated by Bandura et al. (1999) and reflects their confidence in achieving goals or controlling activities]. Previous studies have found that self-efficacy is closely related to the meaning in life. According to Cheng et al. (2021), research shows that self-efficacy is a positive predictor of people’s meaning in life. A recent study found that self-efficacy has a positive impact on the meaning in life and can predict suicidal behaviors in cancer patients (Zhang et al., 2023). Thus, this study proposed hypothesis 2: Self-efficacy is positively associated with the meaning in life for college students.

Individuals evaluate their quality of life based on self-set standards to determine their life satisfaction. Life satisfaction is often used as an indicator of mental health and personal well-being (Diener et al., 1999). Having a sense of purpose and goals in life leads to greater life satisfaction, according to research by Park et al. (2010) and Steger et al. (2009a,b). In addition, a series of empirical studies on college students have demonstrated a positive correlation between life satisfaction and meaning in life (Liu, 2012; Heng et al., 2020). Thus, hypothesis 3 was proposed in this study: Life satisfaction of college students is positively associated with their meaning in life.

### 1.3 Mediating roles of self-efficacy and life satisfaction

In recent years, the impact of sports on an individual’s psychosocial adaptive function has become a prominent topic in sports psychology. Research conducted on college students has indicated that engaging in regular physical exercise can have a positive impact on self-efficacy. This means that students who exercise more frequently tend to possess a higher level of confidence when it comes to achieving their goals (Hou and Yang, 2016; Wang et al., 2022). Furthermore, engaging in physical exercise and building self-confidence can have a mutually beneficial relationship. Physical exercise can increase confidence in achieving goals, which in turn can motivate individuals to continue exercising (Downs, 2014; Zhao et al., 2019). In addition, engaging in physical exercise has been shown to enhance personal satisfaction with life. Previous studies have shown that an individual’s life satisfaction can be influenced by their personality traits, cognitive abilities, behaviors, and social situations (Emmons and Diener, 1985; Kim et al., 2017; Tian et al., 2023). Empirical research supports the role of physical exercise in enhancing life satisfaction. Research shows that regular physical exercise can increase life satisfaction for high school students and long-distance runners (Gül and Küçükibis, 2018; Sato et al., 2018). Research conducted on college students has shown that there is a strong correlation between physical exercise and life satisfaction (Liu et al., 2021). Specifically, college students who engage in sports activities tend to report higher levels of life satisfaction compared to their peers who do not participate in such activities (Arslan and Akkas, 2014). In addition, Self-determination Theory holds that individuals have three basic psychological needs, including autonomy, competence, and relatedness (Deci and Ryan, 2000, 2012), the Basic Psychological Needs Theory (BPNT) emphasizes that satisfying psychological needs is significantly correlated with greater individual happiness and mental health (Ryan et al., 2010). Physical exercise, as a behavior driven by individual intrinsic motivation, can satisfy the autonomy needs of college students, thus promoting self-efficacy and life satisfaction. To sum up, self-efficacy and life satisfaction not only can enhance individual meaning in life but also may be the potential mechanism between physical exercise and meaning in life. Therefore, this study proposed hypotheses 4: Physical exercise is indirectly associated with meaning in life of college students through self-efficacy, and hypothesis 5: Physical exercise is indirectly associated with meaning in life of college students through life satisfaction.

Self-efficacy and life satisfaction may not only be independent as the potential mechanism of physical exercise affecting meaning in life of college students but also have a specific positive correlation between them. In addition, people who possess stronger self-efficacy are more likely to experience greater levels of happiness and contentment in their lives. Social Cognitive Theory emphasizes the role of self-efficacy and believes that it is closely related to individual experience, ability and motivation (Bandura, 1977; Bandura et al., 1999). Therefore, self-efficacy is a positive belief that can affect an individual’s cognition, emotion and strategic choice in the face of stress (Tsang et al., 2012; McKay et al., 2014). In this perspective, previous studies have found that self-efficacy significantly and positively predicts college students’ life satisfaction (Azizli et al., 2015; Tian et al., 2022). The higher the self-efficacy of college students, the more positively they evaluate their quality of life based on personal standards. A recent study found that self-efficacy mediates the relationship between specialized physical exercise and life satisfaction (Tian et al., 2022). Based on the above analysis, this study proposed hypothesis 6: Physical exercise can be indirectly associated with meaning in life of college students through self-efficacy and life satisfaction.

### 1.4 The current study

**Figure 1:**
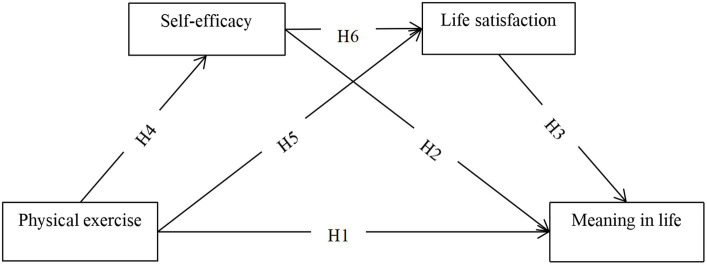
The conceptual model between physical exercise and meaning in life. Hypothesis 1 (H1). Physical exercise will be positively associated with meaning in life. Hypothesis 2 (H2), Self-efficacy will be positively associated with meaning in life. Hypothesis 3 (H3), Life satisfaction will be positively associated with meaning in life. Hypothesis 4 (H4), Self-efficacy will mediate the relationship between physical exercise and meaning in life. Hypothesis 5 (H5), Life satisfaction will mediate the correlation between physical exercise and meaning in life. Hypothesis 6 (H6). Physical exercise can indirectly associated with moaning in life through the chain mediation of self-efficacy and life satisfaction.

College students’ psychological and social adaptation benefit greatly from a meaningful life. Therefore, it is very meaningful to explore the relationship between physical exercise and meaning in life from the physiological and psychological perspectives. Physical exercise is a crucial factor in enhancing one’s meaning in life. This conclusion is based on theoretical and empirical research. Despite the importance of physical exercise for college students, there are few studies exploring how it impacts their meaning in life. To address this gap, this study created a chain mediation model linking physical exercise, self-efficacy, life satisfaction, and meaning in life (as shown in Figure 1). Through this model, we hope to shed light on the complex relationships between these variables and offer insights for enhancing meaning in life of college students. On the one hand, theoretically, our findings can promote the application of Self-determination Theory, Social Cognitive Theory and the theory of mood-enhancing benefits of exercise in the field of meaning in life. On the other hand, in practice, this study combines the physical exercise concerned by sports psychology with meaning in life concerned by positive psychology, analyzes and explains the importance of physical exercise from the psychological level, changes the public’s disdain for physical exercise in the past, and helps to promote the formulation and implementation of “Sport for All” related policies.

## 2 Methods

### 2.1 Participants

A total of 3, 196 college students from North, Central, and East China were recruited to participate in the survey by stratified sampling method. After excluding participants whose grades were non-college students and whose ages were non-college students, the remaining 3, 089 participants were included in the analysis, with an average age of 20.34 ± 1. 29. Of these, 58.1% were female and 41.9% were male. The sample included 1, 406 freshmen (45.5%), 928 sophomores (30.0%), 576 juniors (18.6%), and 179 seniors (5.8%).

### 2.2 Measures

#### 2.2.1 Physical exercise

Physical exercise was measured using the Physical Activity Rating Scale-3 (PARS-3) (Liang, 1994), which consists of three dimensions: exercise intensity, exercise duration, and exercise frequency. Each dimension has only one question (e.g., “How intense is your physical exercise?”). Each question has five options and is scored from 1 to 5. The total score is calculated using the formula: Physical Exercise Level = Exercise Intensity × (Exercise Duration - 1) × Exercise Frequency. The score ranges from 0 to 100, with higher scores indicating higher levels of physical exercise. In this study, the Cronbach’s α was 0. 71.

#### 2.2.2 Life satisfaction

Life satisfaction was measured by the Satisfaction with Life Scale (SWLS) (Pavot and Diener, 1993). It is used to assess individual evaluation of their overall life quality and satisfaction, which comprises 5 items worded as statements (e.g., “In general, I feel I have achieved the important things I want in life.”). Respondents assessed each statement on a 7-point Likert scale, with values ranging from 1 = Strongly disagree to 7 = Strongly agree. Higher scores indicate a higher level of life satisfaction. The Chinese-translated version used in this study has been validated in Chinese populations and has shown good reliability and validity (Xiong and Xu, 2009). The Cronbach’s α in this study was 0. 94.

#### 2.2.3 Self-efficacy

Self-efficacy was measured using the General Self-Efficacy Scale (GSES), which assesses individual subjective evaluation of their confidence in their abilities or ability to perform a specific behavior. The Chinese version revised by Caikang et al. (2001) was used in this study, which comprises three items (e.g., “I can always solve problems if I try hard enough.”). Respondents answered each item on a 4-point Likert scale, ranging from 1 = Not at all true to 4 = Very true, with higher scores indicating stronger self-efficacy. The Cronbach’s α for this questionnaire in the present study was 0. 88.

#### 2.2.4 Meaning in life

Meaning in life was measured using the Meaning in Life Questionnaire (MILQ), which consists of two dimensions: Presence (MIL-P; perceived meaning) and Search (MIL-S; search for meaning) (Steger et al., 2006). The Presence dimension primarily assesses an individual’s perception of the meaning in life. There are five items (e.g., “I understand the meaning of my life.”). “My life has no clear purpose” is reverse-scored. The Search dimension primarily measures an individual’s motivation to seek meaning in life. There are also five items (e.g., “I am searching for meaning in my life.”). Respondents answered all 10 items on a 7-point Likert scale, ranging from 1 = Not at all true to 7 = Very true. Higher scores indicate a more excellent perception of meaning in life. The Chinese translation of the MIL was used in this study (Chen et al., 2015), with a Cronbach’s α of 0. 87.

### 2.3 Data analysis

SPSS 25.0 and SPSS macro-PROCESS were used to analyze the data. Harman’s single-factor analysis was first performed to test for common method bias. Descriptive statistics were then utilized, and the reliability of the scales was assessed using Cronbach’s Alpha coefficient. Pearson correlation coefficients were calculated to examine the relationships between variables. Finally, PROCESS (model 6) was employed to test for chain mediation relationships among self-efficacy, life satisfaction, physical exercise, and meaning in life.

## 3 Results

### 3.1 Common method bias tests

Harman’s single-factor test was conducted in this study to measure common method bias (Podsakoff et al., 2003). The results revealed that there were six factors with eigenvalues greater than 1. The first factor accounted for only 37. 34% of the variance, below the critical threshold of 40%. Therefore, it can be concluded that there is no significant issue of common method bias in this study.

### 3.2 Descriptive statistics and correlations

Table 1 presents the results of the descriptive statistics and correlation analysis in this study. The results indicated that physical exercise positively correlated with self-efficacy, life satisfaction, and meaning in life. These results suggested significant correlations among the variables, providing a solid foundation for subsequent mediation analysis.

**Table 1:** Descriptive statistics and correlations.

Variables	M	SD	α	1	2	3
1. PE	18.25	20.64	0.71			
2. SE	24.44	5.92	0.94	0.20^***^		
3. LS	21.49	6.01	0.88	0.10^***^	0.53^***^	
4. MIL	48.62	8.81	0.87	0.14^***^	0.46^***^	0.55^***^

*N* = 3,089. PE, physical exercise; SE, self-efficacy; LS, life satisfaction; MIL, meaning in life.

^***^ *p* < 0.001.

### 3.3 The chain mediating effects analysis

**Figure 2:**
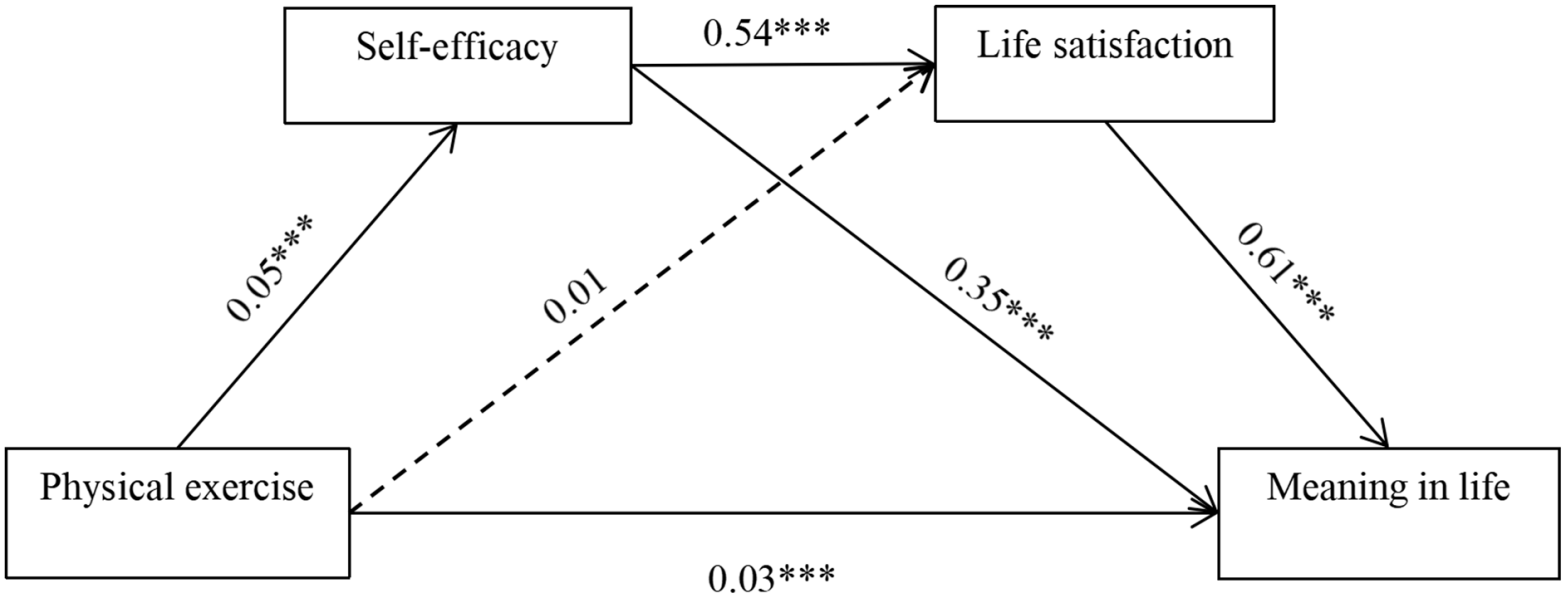
The chain mediation effect of self-efficacy and life satisfaction.

SPSS macro-PROCESS (model 6) was used to analyze the chain mediation effects of self-efficacy and life satisfaction between physical exercise and meaning in life. Controlling for age, gender, and grade level, the results of the study (detailed results in Table 2; Figure 2) indicated that gender was positively associated with meaning in life (*β* = 1. 28, *p* < 0.001) and life satisfaction (*β* = 1.01, *p* < 0.001), and a significant negative predictive effect on self-efficacy (*β* = −1.12, *p* < 0.001). Additionally, physical exercise positively associated with meaning in life (*β* = 0.07, *p* < 0.001), supporting H1. Regarding the mediating role between self-efficacy, life satisfaction, physical exercise, and meaning in life, the results revealed that self-efficacy substantially predicts meaning in life (*β* = 0.35, *p* < 0.001), and life satisfaction also positively associated with meaning in life (*β* = 0.61, *p* < 0.001), supporting H2 and H3. Furthermore, the results showed that physical exercise significantly predicts self-efficacy (*β* = 0.05, *p* < 0.001), H4 was supported. However, the study did not find a significant association between physical exercise and life satisfaction, H5 was not supported. After entering both self-efficacy and life satisfaction into model, the residual direct effect was significant (*β* = 0.03, *p* < 0.001). Moreover, the chain mediating effects of self-efficacy and life satisfaction in the relationship between physical exercise and meaning in life were confirmed (*β* = 0.02, *p* < 0.001). The results for chain mediation showed that self-efficacy and life satisfaction chainly mediated the relationship between physical exercise and meaning in life of college students, thus supporting H6. In addition, based on the educational level of the participants, this study examined the chain mediating effect model in different grades. The results showed that the mediating effect held true in all models with different levels of education, and did not differ from the total model, except the direct effect of physical exercise on the meaning in life was not significant in the juniors (*β* = 0.01, *p* > 0.05) and seniors (*β* = 0.05, *p* > 0.05).

**Table 2:** Regression analysis of the relationship between physical exercise and meaning in life.

Outcome	Predictor	*R* ^2^	*F*	*β*	*t*	95% Boot CI
MIL	Age	0.02	19.03^***^	0.43	2.32^*^	[0.07, 0.79]
	Gender			1.28	3.69^***^	[0.60, 1.97]
	Grade			−0.38	−1.45	[−0.89, 0.13]
	PE			0.07	8.34^***^	[0.05, 0.08]
SE	Age	0.05	38.65^***^	0.18	1.46	[−0.06, 0.42]
	Gender			−1.12	−4.87^***^	[−1.57, 0.67]
	Grade			−0.23	−1.35	[−0.57, −0.11]
	PE			0.05	8.38^***^	[0.04, 0.06]
LS	Age	0.28	244.23^***^	0.14	1.31	[−0.07, 0.35]
	Gender			1.01	4.96^***^	[0.61, 1.41]
	Grade			−0.33	−2.16^*^	[−0.63, −0.03]
	PE			0.01	1.46	[0.00, 0.02]
	SE			0.54	34.14^***^	[0.51, 0.57]
MIL	Age	0.35	277.20^***^	0.22	1.45	[−0.08, 0.51]
	Gender			1.43	5.00^***^	[0.87, 1.99]
	Grade			−0.02	−0.08	[−0.44, 0.40]
	PE			0.03	4.86^***^	[0.02, 0.05]
	SE			0.35	13.49^***^	[0.30, 0.40]
	LS			0.61	24.42^***^	[0.56, 0.66]

*N* = 3,089. PE, physical exercise, SE, self-efficacy, LS, life satisfaction, MIL, meaning in life. Gender, age, grade are the control variables. PE is an independent variable, and MIL is a result variable. This table presents the results of the multiple hierarchical regression analysis of PE, SE, LS and MIL. Boot confidence interval (CI) upper limit (ULCI), and boot CI lower limit (LLCI) are indicative of the standard deviation (SD) of the indirect effect evaluated through the deviation correction percentile Bootstrap approach and the upper and lower limits of the respective 95% confidence intervals (CIs). CI, confidence interval. ^*^p < 0.05; ^**^p < 0.01; ^***^p < 0.001.

To further examine the magnitudes of the mediation effects in each pathway, this study employed bias-corrected bootstrap tests derived from 5, 000 samples. The 95% confidence intervals did not include zero, indicating significant effects. The detailed results are shown in Table 3 and Figure 2. The total effect of physical exercise on meaning in life was 0.069, with a direct effect of 0.033. The total indirect effect was 0.036, accounting for 51.82% of the total effect. The total mediation effect consisted of three indirect pathways: Pathway 1: physical exercise → self-efficacy → meaning in life (0.016); Pathway 2: physical exercise → life satisfaction → meaning in life (0.004); and Pathway 3: physical exercise → self-efficacy → life satisfaction → meaning in life (0.015). As shown in Table 3, for Pathway 2, the 95% confidence interval includes zero, indicating that this indirect effect is insignificant. The other two pathways have 95% confidence intervals that did not include zero, showing significant indirect effects for these two pathways, which further validate H4 and H6. Comparing the confidence intervals of Pathways 1 and 3 with Pathway 2, it can be inferred that the indirect effects of Pathways 1 and 3 are significantly different. In contrast, the inclusion of zero in Pathway 2 suggested that there is no significant difference between Pathways 1 and 3 in terms of indirect effects.

**Table 3:** Self-efficacy and life satisfaction in the mediation effect analysis.

	Indirect effects	Boot *SE*	Boot LLCI	Boot ULCI	Relative mediation effect
Total indirect effect	0.0355	0.0052	0.0254	0.0460	51.82%
Indirect effect 1	0.0160	0.0024	0.0116	0.0210	23.36%
Indirect effect 2	0.0043	0.0031	−0.0016	0.0106	
Indirect effect 3	0.0152	0.0021	0.0112	0.0195	22.19%
Compare 1	0.0117	0.0039	0.0039	0.0192	
Compare 2	0.0008	0.0019	−0.0028	0.0046	
Compare 3	−0.0108	0.0037	−0.0180	−0.0035	

*N* = 3,089. The direct and indirect effects of physical exercise on meaning in life are shown in this table. Boot SE, Boot LLCI, and Boot ULCI refer, respectively, to the standard error and the lower and upper limits of the 95% confidence interval of the indirect effects estimated by the percentile bootstrap method with deviation correction. Indirect effect 1: physical exercise → self-efficacy → meaning in life; indirect effect 2: physical exercise → life satisfaction → meaning in life; indirect effect 3: physical exercise → self-efficacy → life satisfaction → meaning in life. Compare 1: indirect effect 1–indirect effect 2; compare 2: indirect effect 1–indirect effect 3; compare 3: indirect effect 2–indirect effect 3.

In summary, the research findings demonstrated that physical exercise can indirectly predict an individual’s meaning in life through self-efficacy and a chain mediation pathway involving self-efficacy and life satisfaction.

## 4 Discussion

This study used a chain mediation model to examine the relationship between college students’ self-efficacy, life satisfaction, physical exercise, and meaning in life. The study found that engaging in physical exercise can lead to greater self-efficacy, life satisfaction, and ultimately generate higher meaning in life. Additionally, the study revealed that self-efficacy mediates the relationship between physical exercise and meaning in life. However, the path of mediation through life satisfaction does not hold significant importance. The findings of this study can offer evidence of how physical exercise affects the meaning in life. It can also provide empirical support for enhancing the meaning in life for college students, decreasing suicidal tendencies, and promoting better mental health among them.

### 4.1 The impact of physical exercise on meaning In life

This study found that physical exercise is positively associated with meaning in life of college students, confirming H1. This study replicated previous research findings (Takkinen et al., 2001; Liu, 2012; Xia et al., 2023), supporting the idea that physical exercise has a positive impact on the meaning in life of college students. Studies have shown that exercise not only benefits athletes physically but can also have a positive impact on their emotional well-being (Anderson and Brice, 2011; Rebar et al., 2015). In turn, this can greatly enhance college students’ sense of purpose and meaning in life (Abeyta et al., 2015). College students should possess not only professional knowledge and technology but also a healthy mind and body to become a driving force for the country’s future development. The study suggested that college students should prioritize physical exercise for overall well-being.

### 4.2 The mediating role of self-efficacy

This study found that self-efficacy indirectly links physical exercise to meaning in life of college students, confirming research on H2 and H4. Studies have shown that college students can improve their confidence in achieving goals and self-efficacy through physical exercise (Hou and Yang, 2016; Wang et al., 2022). The findings of this study also uncovered this particular connection. Previous studies have shown that boosting self-efficacy can aid college students in understanding the significance of their lives (Cheng et al., 2021). This conclusion has also been observed in research conducted on psychiatric patients (Zhang et al., 2023). In the current study, our results not only confirm previous findings but also establish a logical link between physical exercise and improved meaning in life of college students. Engaging in physical exercise can boost self-efficacy and provide a sense of accomplishment and control over physical activities. Self-efficacy is a concept that highlights the importance of an individual’s self-assessment of their ability to attain their objectives. Both domestic and foreign scholars emphasize direction and purpose when explaining the meaning in life (Liu, 2012). The practice of physical exercise can improve self-efficacy, leading to increased confidence in achieving personal goals and a greater sense of meaning in life. It can be seen that physical exercise can enhance meaning in life of college students by improving their self-efficacy.

### 4.3 The mediating role of life satisfaction

This study did not find evidence to support the mediating effect of life satisfaction on the relationship between physical exercise and meaning in life, therefore H5 was not confirmed. This result suggests that physical exercise may indirectly impact life satisfaction by shaping other positive psychological traits in individuals. This explanation aligns with prior research findings as well. First of all, previous research have shown that physical exercise does not directly impact life satisfaction but can enhance it indirectly through improved self-control, mental health, and social support (Dai and Yao, 2012; Zhou et al., 2023). According to Zhou et al. (2023), engaging in physical exercise can improve self-control, leading to a reduction in psychological distress and an increase in life satisfaction. Engaging in physical exercise can also enhance one’s self-efficacy and perceived social support, ultimately leading to greater individual satisfaction with life (Dai and Yao, 2012).

Secondly, various factors such as personality, cognition, behavior, and social context affect life satisfaction, a subjective evaluation of life quality (Emmons and Diener, 1985; Tian et al., 2023). While physical exercise is an external behavioral factor, it may not immediately affect the life satisfaction of college students like cognitive and personality traits. Positive psychological traits were shaped to achieve it, which may take a long time, so it cannot predict college students’ life satisfaction directly given the cross-sectional nature of the data. To test this hypothesis, future studies can use a longitudinal design. Finally, according to a recent survey on college students’ physical exercise, there is a lack of proper supervision and management (Li et al., 2021), which has resulted in college students not reaching their ideal level of physical exercise. This study indicates that the mediation role of life satisfaction was not significant, which may be due to the lack of physical exercise in daily life for college students. They tend to choose other ways, such as recreational activities, to reduce pressure and improve personal life satisfaction, which failed to reflect the value of physical exercise in improving life satisfaction.

### 4.4 The chain mediating roles of self-efficacy and life satisfaction

According to this study, self-efficacy and life satisfaction of college students mediate the relationship between physical exercise and their meaning in life. In simpler terms, physical exercise indirectly affects college students’ meaning in life through their self-efficacy and life satisfaction. H3 and H6 have been confirmed. Physical exercise can enhance self-efficacy of college students, leading to improved subjective evaluation of current quality of life and perception of meaning in life. As mentioned above, in Social Cognitive Theory, self-efficacy is considered to be a positive belief (Tsang et al., 2012; McKay et al., 2014), it helps individuals face different pressures and enhances their positive experiences, such as positive emotions and life satisfaction (Azizli et al., 2015; Tian et al., 2022). In addition, the theory of mood-enhancing benefits of exercise emphasizes that physical exercise can bring positive emotional experience to individuals (Anderson and Brice, 2011), so that the individual’s self-efficacy is enhanced, thus promoting their life satisfaction. The findings of this study confirm this idea, they also support that there is a complex relationship between participating physical exercise and finding meaning in life. It could be used to help college students find more meaning in their lives and guide interventions to help them. Additionally, it could inform the development of policies related to this topic.

On the whole, this study reveals the potential psychological mechanism of physical exercise affecting meaning in life of college students: Physical exercise have an impact on college students’ meaning in life through the chain mediating role of self-efficacy and life satisfaction. This result confirms the mechanism of the influence of exercise on individual psychology, and enriches the research findings in the fields related to sports psychology and positive psychology. On the one hand, it suggests that researchers should combine the study of sports and psychology. On the other hand, it suggests that college administrators should attach importance to the daily physical exercise of college students, pay attention to the publicity of the positive impact of physical exercise, and enhance the meaning in life of college students, promote and maintain their mental health.

## 5 Implications, limitations and future research

The results of this study provide significant theoretical and unique empirical contributions. Previous studies have found that physical exercise enhances meaning in life. However, there are no studies investigating its mechanism in college students. This study investigated how physical exercise affects meaning in life of college students by constructing a chain mediation model to identify multiple paths. The study found that physical exercise is positively associated with meaning in life for college students, both directly and indirectly through self-efficacy and life satisfaction, which enhances existing literature and provides fresh insights to enhance meaning in life of college students. Moreover, from an applied perspective, this study confirms that physical exercise is crucial in improving the meaning in life among college students. Encouraging college students to engage in physical exercise can positively impact their meaning in life by boosting their self-efficacy and life satisfaction. Therefore, it is important to guide them in building their self-confidence and hope for the future through physical activity. This can lead to increased life satisfaction and self-efficacy, ultimately promoting a greater meaning in their lives. Based on the findings, it seems that physical exercise does not indirectly impact the meaning in life for college students through life satisfaction. It indicates that intermittent physical exercise may not necessarily improve their meaning in life. Therefore, it is important to maintain a regular exercise routine as it can contribute to an overall healthier lifestyle and may indirectly improve one’s meaning in life.

However, the current study had some limitations. Firstly, it used a cross-sectional design to examine the connection between physical exercise and meaning in life and found that physical exercise had a direct or indirect impact on meaning in life of college students. Since cross-sectional studies are prone to instability and lack of continuity, the causal relationship revealed in this study cannot be verified repeatedly. To obtain more stable and convincing research results, future studies should conduct experiments to reveal the causal relationship between physical exercise and meaning in life. Longitudinal follow-up surveys should also be designed to explore any possible causal relationship between these variables.

Secondly, this study examines physical exercise among college students, including intensity, time, and frequency. Previously, numerous researchers have examined the effects of physical exercise on different variables within each dimension. However, this study specifically examines how physical exercise overall impacts the meaning in life for college students. Future studies may delve into the different dimensions of physical exercise and their effects on meaning in life, resulting in more comprehensive and detailed research findings.

Finally, recent studies have shown that engaging in physical exercise can have numerous benefits for our health. It can help prevent cardiovascular diseases, reduce the risk of neurodegenerative diseases and brain lesions, regulate brain circuits by controlling the release of neurotransmitters, and even improve cognitive abilities (Blair and Brodney, 1999; M. Stranahan et al., 2012; Allard et al., 2017). The results provided are enough to demonstrate the influence of physical exercise on an individual’s brain function. As a result, future studies could begin by examining cognitive nerves to investigate the variations in functional brain activation patterns or functional connectivity among individuals with varying degrees of physical exercise (Bray et al., 2021). They can use tools like fNIRS and fMRI to analyze the physiological mechanisms that affect an individual’s meaning in life from a physiological perspective (Dudar et al., 1979; Allard et al., 2017).

## 6 Conclusion

In conclusion, it was discovered that physical exercise is a crucial factor for meaning in life. Additionally, current findings suggested that the relationship between physical exercise and meaning in life of college students may be mediated by self-efficacy and life satisfaction. These findings offer a deeper understanding of how and why physical exercise may put college students at increased the meaning in life, and provide an empirical evidence and valuable intervention insights for future research on the topic.

## Data availability statement

The raw data supporting the conclusions of this article will be made available by the authors, without undue reservation.

## Ethics statement

Ethical review and approval was not required for the study on human participants in accordance with the local legislation and institutional requirements. Written informed consent from the patients/participants or patients/participants’ legal guardian/next of kin was not required to participate in this study in accordance with the national legislation and the institutional requirements.

## Author contributions

YG: Data curation, Investigation, Writing – original draft, Project administration. KQ: Conceptualization, Formal analysis, Methodology, Writing – original draft, Resources. YY: Writing – review & editing. LW: Conceptualization, Writing – review & editing. FX: Writing – review & editing. QZ: Writing – review & editing. XH: Writing – review & editing. YZ: Writing – review & editing. BH: Writing – review & editing. QH: Writing – review & editing, Writing – review & editing. CG: Writing – review & editing, Supervision. JZ: Writing – review & editing, Funding acquisition.
